# Whole genome sequencing data from pedigrees suggests linkage disequilibrium among rare variants created by population admixture

**DOI:** 10.1186/1753-6561-8-S1-S44

**Published:** 2014-06-17

**Authors:** Tao Feng, Xiaofeng Zhu

**Affiliations:** 1 Department of Epidemiology and Biostatistics, Case Western Reserve University, Wolstein Research Building, 2103 Cornell Road, Cleveland, OH 44106, USA

## Abstract

Next-generation sequencing technologies have been designed to discover rare and *de novo *variants and are an important tool for identifying rare disease variants. Many statistical methods have been developed to test, using next-generation sequencing data, for rare variants that are associated with a trait. However, many of these methods make assumptions that rare variants are in linkage equilibrium in a gene. In this report, we studied whether transmitted or untransmitted haplotypes carry an excess of rare variants using the whole genome sequencing data of 15 large Mexican American pedigrees provided by the Genetic Analysis Workshop 18. We observed that an excess of rare variants are carried on either transmitted or nontransmitted haplotypes from parents to offspring. Further analyses suggest that such nonrandom associations among rare variants can be attributed to population admixture and single-nucleotide variant calling errors. Our results have significant implications for rare variant association studies, especially those conducted in admixed populations.

## Background

Next-generation sequencing technologies have become a major tool for identifying disease-associated rare variants [1]. Many statistical methods have been developed to test for association between rare variants and complex traits using next-generation sequencing data [2-8]. Most statistical methods for rare variant association testing either do not address rare variant calling errors or indirectly assume that rare variants are correctly called. We studied the distribution of rare variants in transmitted and untransmitted haplotypes from parents to their offspring in nuclear families using whole genome sequencing data from15 large Mexican American pedigrees provided by the Genetic Analysis Workshop 18 (GAW18). We observed an excess of rare variants falling on either transmitted or nontransmitted haplotypes from parents to offspring, suggesting linkage disequilibrium (LD) among rare variants and/or single-nucleotide variant (SNV) calling errors.

## Methods

### Data preparation

The GAW18 data includes 464 Mexican American individuals from 16 large pedigrees with half genome sequence data available. Our goal is to study the LD among rare variants using the sequencing data by comparing the number of rare variants on transmitted and untransmitted haplotypes. In our analysis, we excluded all the single-nucleotide polymorphisms (SNPs) with a minor allele frequency (MAF) >0.01. We also excluded these SNPs with (a)a missing genotyping rate >5%; (b) Hardy-Weinberg equilibrium (HWE) test *p *values <0.001; and (c) observed Mendelian errors.

GAW18 also provides hypertension data. We used the hypertension status provided by GAW18, which is based on blood pressure measurements at 4 study exams in the past 20 years. An individual is defined as hypertensive if the individual's systolic blood pressure (SBP)>140, or diastolic blood pressure (DBP)>90, or on antihypertensive medications at one of 4 exams and as normotensive otherwise. If an individual has missing values for all 4 exams, the individual's hypertension status is considered as missing.

### Analysis method

We first selected family trios of "mother, father, and child" from each of the 16 large pedigrees. Among the 16 pedigrees, 1 pedigree does not include any trio who has sequencing data available. Thus, the family trios were all from 15 pedigrees. Similar to the traditional transmission disequilibrium test (TDT), we examine the transmission of a rare variant allele. Because our analysis focuses on those variants with a MAF≤1%, we expect no more than 1 parent to be heterozygous. If both parents are heterozygous for a variant, this variant is excluded from our analysis. Thus, any minor transmitted alleles to an offspring from a parent must fall on the same haplotype because of no recombination. We examine all the rare variants in a gene or a region simultaneously instead of examining 1 variant at a time. Let *m *be the number of trios regardless of an offspring's disease status in the data and *L *be the number of rare variants in a gene or a genomic region. We denote *m*_12 _to be the total number of transmitted minor alleles and *m*_21 _to be the total number of nontransmitted minor alleles across the *L *variants in *m *trios. In this way, *m*_12 _is equal to the total number of rare variants falling in transmitted haplotypes, and *m*_21 _is the total number of rare variants in nontransmitted haplotypes, respectively. If rare variants are randomly distributed in haplotypes (or, equivalently, there is no LD among rare variants), we would expect *m*_12 _=*m*_21_. We can use the TDT statistic $T=\frac{{\left(m_{12}-m_{21}\right)}^{2}}{m_{12}+m_{21}}$ for testing the randomness among the rare variants. The statistic *T *follows a chi square distribution with 1 degree of freedom (DF). When only the trios with affected offspring are included, this test will test the association between rare variants and disease status.

## Results

We applied the proposed methods to the GAW18 sequence data. After quality control, there are 2,749,275 rare SNPs remaining for association analysis. We identified 5 trios with affected offspring and 36 trios with unaffected offspring. Because the number of affected offspring trios is small, we only analyzed the unaffected offspring trios. Because some of the trios were selected from the same pedigrees, we analyzed 15 independent unaffected offspring trios. This was done by randomly selecting 1 family trio if multiple family trios were available for a pedigree. We grouped SNPs into genes or regions according to the Ensembl software (http://www.ensembl.org). As a result, we had 38,091 genes and regions. The average number of SNPs in a gene or a region was 92.

**Figure 1:**
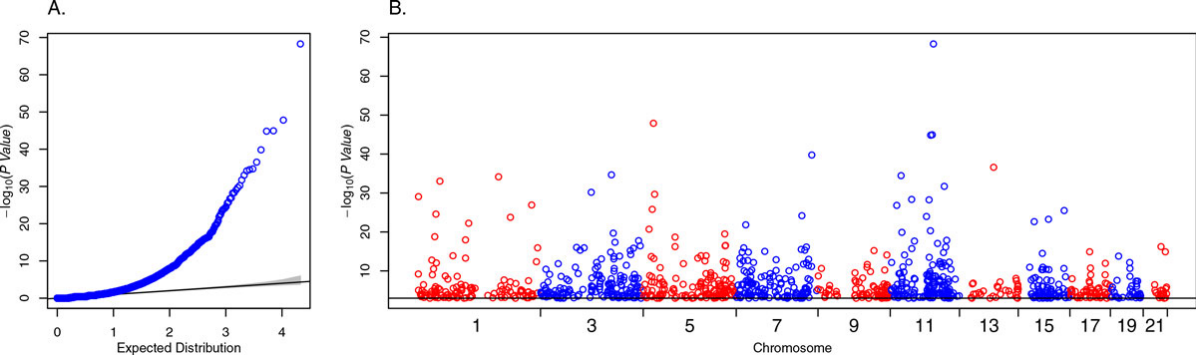
**A, Q-Q plot of −log10(*p *value) based on randomly selected 15 independent nuclear families;B, Manhattan plot of the genes or regions with −log10(*p *value)>3 based on the15 nuclear families**. The horizontal line represents −log10(*p *value)=3

Figure 1A presents the Q-Q plot of −log10(*p *value) for all the genes or regions across the genome and we observed a substantial inflation of the test statistic, suggesting rare variants are not randomly distributed on transmitted and nontransmitted haplotypes. We examined where these significant genes were located in the genome using the Manhattan plot (Figure 1B). We observed that these significant genes are distributed across the genome evenly rather than clustered in a few regions, suggesting our test is not testing for linkage. We then examined the 20 most significant genes, which are presented in Table 1. Among these top 20 genes, 18 genes have more rare variants on nontransmitted than on transmitted haplotypes and 2 are the other way round. We hypothesized that the excess of rare variants on nontransmitted haplotypes is probably caused by SNV calling errors. The reason is that when a rare variant is observed in an offspring, it should also be observed in 1 of the offspring's parents. Otherwise Mendelian error examination will filter out this variant. However, Mendelian error examination will not filter out any SNV calling errors on nontransmitted haplotypes unless all grandparents are in pedigrees and their sequencing data are available. Thus, an excess of rare variants on nontransmitted haplotypes may be expected. However, this does not explain the excess of rare variants on transmitted haplotypes for the 2 genes: *CD247 *and *KIF1B*. Consequently, we examined whether the excess of rare variants was caused by some specific families. In fact, this is true, as we observed that these significant *p *values could be attributed to a small number of transmitted or nontransmitted haplotypes for each gene (Table 1). We then tried to answer why these haplotypes carry significantly more rare variants. Specifically, we examined two genes, *CD247 *and *KIF1B*, which have an excess of rare variants on transmitted compared to nontransmitted haplotypes. Because the transmitted rare variants were present in offspring and 1 of 2 parents, these variants are less likely to be mistakenly called. For the *CD247 *gene, we identified 1 transmitted haplotype carrying 175 rare variants. We searched the 1000 Genomes Project database (http://browser.1000genomes.org/) and identified 170 of the 175 variants as present in the 1000 Genomes Project database. Among these 170 variants, 154 variants are present only in African samples; the other 16 variants are present in Africans and in other ethnic populations in the 1000 Genomes Project database. Similar results were observed for the *KIF1B *gene. Thus, our result suggests that the excess of rare variants in haplotypes, or the LD among the rare variants, is caused by population admixture with African ancestry populations.

**Table 1:** P values of the top 20 genes based on 15 nuclear families

Chr	Gene	# of rare variants transmitted	# of rare variants non transmitted	*p *Value	# of trios contributing most statistical evidence	# rare variants transmitted in the most contributing trios	# of rare variants nontransmitted in the most contributing trios
11	DLG2	279	876	4.45 × 10^−69^	4	44	578

5	CDH18	117	473	1.23 × 10^−48^	2	12	272

11	RP11-179A16.1.1	122	466	1.11 × 10^−45^	4	30	376

11	ODZ4	73	372	1.33 × 10^−45^	4	16	290

7	CNTNAP2	200	570	1.47 × 10^−40^	2	21	309

13	PCDH9	73	329	2.47 × 10^−37^	1	13	217

3	STAG1	31	233	1.75 × 10^−35^	3	3	207

11	NELL1	114	393	2.93 × 10^−35^	3	26	232

1	CD247	185	12	6.58 × 10^−35^	1	175	2

1	OSBPL9	19	197	9.20 × 10^−34^	1	0	166

11	GRIA4	28	212	1.56 × 10^−32^	3	6	156

3	EPHA6	151	430	5.53 × 10^−31^	6	95	366

5	CDH12	95	332	1.88 × 10^−30^	2	15	190

1	KIF1B	199	28	7.44 × 10^−30^	1	180	1

11	RP11-124G5.3.1	16	168	3.83 × 10^−29^	1	1	146

11	UVRAG	35	210	5.09 × 10^−29^	4	5	182

1	DISC1	60	253	1.04 × 10^−27^	3	27	217

11	TEAD1	40	213	1.49 × 10^−27^	2	10	161

5	RP11-454P21.1.1	24	174	1.57 × 10^−26^	3	4	158

15	RP11-387D10.2.1	12	145	2.55 × 10^−26^	1	0	132

## Discussion

It has been suggested that rare variants are likely independent in general [6]. However, our analysis suggests that substantial LD among rare variants could be introduced by population admixture. Wang and Zhu [9] suggested that there are substantial genotype calling errors, especially for rare and *de novo *variants, in whole sequencing data. But genotype calling errors are unable to explain the excess of rare variants carried by a few haplotypes in this data. When association tests for rare variants are conducted in admixed populations such as African Americans and Mexican Americans, the LD among rare variants created by population admixture can generate false-positive findings. Our results also suggest that even the TDT may not overcome this problem if multiple rare variants are analyzed together.

## Conclusions

In summary, our analysis indicates that substantial LD among rare variants can be created by population admixture and by genotype calling errors. Novel statistical approaches for rare variant association analysis are required to account for the LD among the rare variants because of either population admixture or genotype calling errors. Family data have been suggested as having many statistical advantages in detecting rare disease variants [4,10] and may help address these problems.

## Competing interests

There is no competing interest among the authors.

## Authors' contributions

XZ designed the overall study, TF conducted statistical analyses, TF and XZ drafted the manuscript. All authors read and approved the final manuscript. We thank the 2 anonymous reviewers and Dr. Heather Cordell for their constructive comments, which lead to the substantial improvement of the manuscript.

## References

[B1] A map of human genome variation from population-scale sequencing Nature 2010 467 1061 1073 1000 Genomes Project Consortium 10.1038/nature09534 20981092 PMC3042601

[B2] Bansal V Libiger O Torkamani A Schork NJ Statistical analysis strategies for association studies involving rare variants Nat Rev Genet 2010 11 773 785 10.1038/nrg2867 20940738 PMC3743540

[B3] Browning SR Browning BL Rapid and accurate haplotype phasing and missing-data inference for whole-genome association studies by use of localized haplotype clustering Am J Hum Genet 2007 81 1084 1097 10.1086/521987 17924348 PMC2265661

[B4] Feng T Elston RC Zhu X Detecting rare and common variants for complex traits: sibpair and odds ratio weighted sum statistics (SPWSS, ORWSS) Genet Epidemiol 2011 35 398 409 10.1002/gepi.20588 21594893 PMC3114642

[B5] Han F Pan W A data-adaptive sum test for disease association with multiple common or rare variants Hum Hered 2010 70 42 54 10.1159/000288704 20413981 PMC2912645

[B6] Li B Leal SM Methods for detecting associations with rare variants for common diseases: application to analysis of sequence data Am J Hum Genet 2008 83 311 321 10.1016/j.ajhg.2008.06.024 18691683 PMC2842185

[B7] Neale BM Rivas MA Voight BF Altshuler D Devlin B Orho-Melander M Kathiresan S Purcell SM Roeder K Daly MJ Testing for an unusual distribution of rare variants PLoS Genet 2011 7 e1001322 10.1371/journal.pgen.1001322 21408211 PMC3048375

[B8] Wu MC Lee S Cai T Li Y Boehnke M Lin X Rare variant association testing for sequencing data using the sequence kernel association test (SKAT) Am J Hum Genet 2011 89 82 93 10.1016/j.ajhg.2011.05.029 21737059 PMC3135811

[B9] Wang H Zhu X De novo mutations discovered in 8Mexican American families through whole genome sequencing BMC Proc 2014 8 suppl 2 S24 10.1186/1753-6561-8-S1-S24 PMC4143763 25519376

[B10] Zhu X Feng T Li Y Lu Q Elston RC Detecting rare variants for complex traits using family and unrelated data Genet Epidemiol 2010 34 171 187 10.1002/gepi.20449 19847924 PMC2811752

